# Electrochemistry and Photoredox Catalysis: A Comparative Evaluation in Organic Synthesis

**DOI:** 10.3390/molecules24112122

**Published:** 2019-06-05

**Authors:** Rik H. Verschueren, Wim M. De Borggraeve

**Affiliations:** Department of Chemistry, Molecular Design and Synthesis, KU Leuven, Celestijnenlaan 200F, box 2404, 3001 Leuven, Belgium; rik.verschueren@kuleuven.be

**Keywords:** electrosynthesis, electrocatalysis, photocatalysis, photochemistry, electron transfer, redox catalysis, radical chemistry, organic synthesis, green chemistry

## Abstract

This review provides an overview of synthetic transformations that have been performed by both electro- and photoredox catalysis. Both toolboxes are evaluated and compared in their ability to enable said transformations. Analogies and distinctions are formulated to obtain a better understanding in both research areas. This knowledge can be used to conceptualize new methodological strategies for either of both approaches starting from the other. It was attempted to extract key components that can be used as guidelines to refine, complement and innovate these two disciplines of organic synthesis.

## 1. Introduction

Both electrochemistry as well as photoredox catalysis have gone through a recent renaissance, bringing forth a whole range of both improved and new transformations previously thought impossible. In their growth, inspiration was found in older established radical chemistry, as well as from cross-pollination between the two toolboxes. In scientific discussion, photoredox catalysis and electrochemistry are often mentioned alongside each other. Nonetheless, no review has attempted a comparative evaluation of both fields in organic synthesis. Both research areas use electrons as reagents to generate open-shell radical intermediates. Because of the similar modes of action, many transformations have been translated from electrochemical to photoredox methodology and vice versa. This reciprocity is very interesting as it provides the ideal study ground for analysis and comparison of both fields. In this review, we look where those research domains interlock and where they differ. Analogies will be drawn between both methods to obtain a better understanding in both areas of research. In an effort to do so, this review will be selective. Transformations were selected which had reports of great similarity to ease comparison. This comparative evaluation can serve as a guidance tutorial review and as an introduction to the fields of electrochemistry and photoredox catalysis in organic synthesis. More rigorous reviews of the literature can be found in the following accounts and references therein [1,2,3,4,5,6,7,8,9,10,11,12]. The present study serves to provide a summary of synthetic transformations that can be performed by electrochemistry as well as photoredox catalysis to gain a better understanding of both. Key features are extracted that can be used as guidelines to formulate new ideas.

In the following section we will display 10 synthetic transformations that have been performed by both electro- and photoredox catalysis and one photoelectrochemical method integrating these two. Schematic presentations of the conditions, the respective mechanisms (as proposed in literature) and a selected scope will assist in a side by side comparison of the two synthetic methodologies for the same transformation.

## 2. Side by Side Comparison of Synthetic Methodologies

### 2.1. Dehydrogenative Lactonization of C(sp^2^/sp^3^)-H Bonds

**Figure 1:**
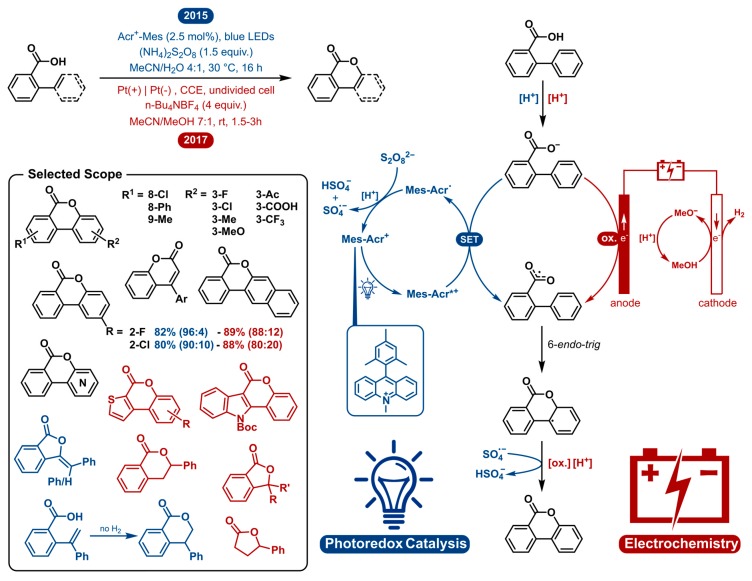
Side by side, a comparative visualization of the photoredox catalyzed and electrochemical dehydrogenative lactonization of C-H bonds regarding reaction conditions, mechanism and product scope. Scope examples shown to work with both methods in black, solely shown by photoredox catalysis in blue and by electrosynthesis in red. CCE = constant current electrolysis, Mes-Acr^+^ = 9-mesityl-10-methylacridinium.

The lactonization of carboxylic acids has evolved as a very convenient approach to integrate lactones as structural subunits in molecules of medicinal and biological interest [13,14,15,16,17,18]. Among the established approaches, direct dehydrogenative C-H/O-H oxidative coupling has gained special interest due to its high atom economy [19,20,21,22,23,24,25,26,27,28]. Pioneering work was reported in 2013 by Martin [19], Wang [20] and Gevorgyan [21], who independently developed the intramolecular oxidative coupling of C(sp^2^)-H bonds of aromatic carboxylic acids using Cu- and Pd-catalysis. In 2015, the Xu group reported a mild, silver catalyzed version of C(sp^2^)-H lactonization [23]. Nonetheless, most of these methods use high temperatures, transition-metal catalysts and strong oxidants. In this comparative segment we will analyze how photoredox catalysis and electrochemistry perform regarding the dehydrogenative lactonization of C(sp^2^/sp^3^)-H bonds (Figure 1).

Gonzalez-Gomez was the first to employ a photoredox catalyst to facilitate the transformation in 2015 [24]. In 2017, Luo disclosed a very similar method using photoredox cobalt co-catalysis and the inclusion of C(sp^2^)-X (X = F, Cl, Br, OMe, SMe) bond cleavage which also yields the lactonization adducts [27]. In 1938, Fichter and coworkers described the first anodic lactonization [29]. They reported the electrosynthesis of 5-methyl phthalide from 2,4-dimethyl benzoic acid. Later, in 2017, Lam and coworkers reported another electrosynthesis of phthalides from aromatic carboxylic acids in combination with aliphatic carboxylic acids [30]. The first dehydrogenative lactonization of biphenyl-2-carboxylic acids by anodic oxidation was reported by Tylor and coworkers in 1957 [31]; however, the electrochemical approach was not the main focus. In 2017 Zeng [28], closely followed in 2018 by Xu [26] and Luo [25], independently reported more elaborate electrochemical dehydrogenative lactonizations of C(sp^2^/sp^3^)-H Bonds. For both strategies we will mainly discuss and compare the work of Gonzalez-Gomez and Zeng in more detail (Figure 1).

Both the photoredox and electrochemical approach omit the use of transition metal catalysts as well as high temperatures. Most of the earlier, transition metal catalyzed procedures use temperatures above 75 °C to overcome the large HOMO-LUMO energy gap needed for the reaction to take place [19,20,21,23]. As can be seen in Figure 1, both the photoredox catalyzed and electrochemical reaction mechanistically operate via single electron oxidation-radical cyclization. The 9-mesityl-10-methylacridinium (Acr-Mes^+^) cation contains one of the most oxidizing triplet states among the organophotocatalysts. Upon irradiation with visible light, the excited photocatalyst generates a carboxylate radical via single electron transfer (SET). The catalyst turnover occurs via reoxidation with a persulfate as the terminal oxidant. In the electrochemical pathway, the same single electron oxidation occurs at the anode by anodic oxidation. At the cathode, hydrogen gas is generated at the same time forming methoxide which can deprotonate the carboxylic acid prior to its oxidation. Following carboxylate radical generation, the lactonization of the C(sp^2^)-H bond happens by way of a 6-*endo*-trig cyclization. Finally, rearomatization by another single electron oxidation occurs. The final oxidant for the photoredox catalyzed reaction is the sulfate radical-anion [SO_4_^•−^] originating from the catalyst regeneration. In the electrosynthesis, again, this happens by anodic oxidation. Comparing both methods, it can be noticed that a common path runs like a thread through the entire mechanism, with the two oxidations being distinct. The advantage with electrosynthesis is that it obviates the need of external oxidants where photoredox catalysis needs a terminal oxidant, even though the strong oxidizing power comes from the photocatalyst. Both methods are uncomplicated and innocuous, having very similar general conditions. An acetonitrile/protic solvent mixture was reported to be the solvent system of choice. Other, similar publications communicate different solvent systems which are also suitable for this transformation, with the common denominator of them performing well in acetonitrile and polar protic solvent systems (TFE, HFIP). Both methods show very similar high yields, though the electrochemical method is generally faster in nature and avoids the use of external oxidants.

Both procedures demonstrate the ability to cyclize a wide range of carboxylic acids. All literature examples on the photoredox catalyzed and electrochemical lactonization cover a broad collective scope. In Figure 1, a selected scope is presented displaying examples that were shown to work with both methods in black, solely shown by photoredox catalysis in blue and by electrosynthesis in red. A wide range of 2-arylbenzoic acids can be cyclized towards the corresponding benzocoumarins. The lactonization of 3′-substituted acids poses a regioselectivity problem. Nonetheless, both methods can cyclize these substrates in good yields with regioselectivities ranging from 80:20 to 96:4, being an improvement over some previous radical lactonization methods [19,21,22]. Comparing the analogous 3′-substituted substrates in the respective scopes, the photocatalytic strategy is slightly more regioselective. Further, heterocyclic substrates and 2-substituted cinnamic acids are reported for both approaches. In 2017, Luo et al. demonstrated a photoredox cobalt co-catalyzed lactonization including a substrate scope with *ortho*-enyl benzoic acids to yield the 5-*exo* adducts and one example that reacted to form the 6-*endo* adduct without hydrogen evolution. The scope of Zeng et al. included the electrochemical dehydrogenative lactonization of C(sp^3^)-H bonds. 2-Benzylbenzoic acids exclusively generated phenyl phthalides without observing C(sp^2^)-H coupling products. Tertiary C(sp^3^)-H bonds could also be formed, and one example of an aliphatic carboxylic acid is given.

In summary, both photoredox catalysis and electrochemistry provide a general and facile access to a multitude of lactones under environmentally benign and mild conditions. The electrosynthesis is preferable when chemical oxidants are to be avoided and fast reactions times are desired. The regioselectivity seems to be superior via the photochemical approach. Finally, both methods have been proven to be scalable on laboratory scale.

### 2.2. Dehydrogenative Lactamization

**Figure 2:**
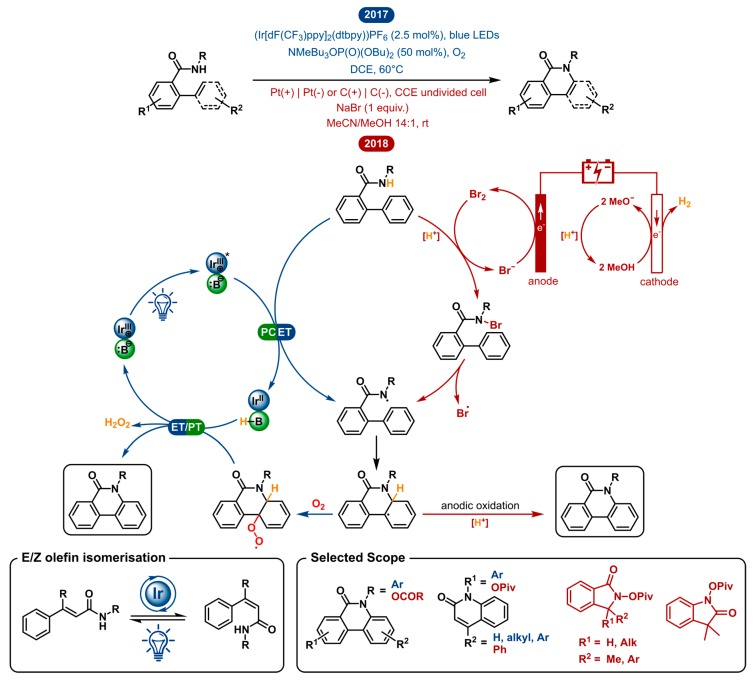
Side by side, comparative visualization of the photoredox catalyzed and electrochemical dehydrogenative lactamization regarding reaction conditions, mechanism and product scope.

Lactams (as well as lactones) serve as a very common subunit in pharmaceutical, medicinal and biological scaffolds. For this reason, synthetic strategies aiming at the synthesis of lactams have been widely explored. Of these methods, a straightforward approach is the direct dehydrogenative C-H/N-H oxidative coupling. In 2017 and 2018 the Hong and Zeng groups, respectively, disclosed a photoredox catalyzed and electrochemical lactamization [32,33]. The following segment aims to illustrate the differences and similarities of both methodologies by taking a closer look at the suggested mechanisms (Figure 2).

Both the photoredox catalyzed and electrochemical approach use the efficacy of nitrogen-centered radicals to establish C-N bond formation. The amidyl radical is key in both, but the generation of the reactive intermediate is distinct. In the photochemical strategy, the cooperation of a weak phosphate base and an excited photocatalyst *Ir^III^ homolytically cleaves the N-H bond of the amide substrate. This happens in a single step through a concerted proton-coupled electron transfer (PCET) generating the key amidyl radical. A similar intermediate is electrochemically generated in three distinct steps. The conjugated MeO^−^ base is generated by cathodic reduction and will first deprotonate the amide substrate. The latter will be trapped by in situ generated bromine to form a nitrogen-bromine bond. Finally, homolysis of the N-Br bond yields the key amidyl radical. The umpolung reactivity of the open-shell species compared to the classical nucleophilic nature of the closed-shell nitrogen species is pertinent for this reaction [34]. The electrophilicity of the amidyl radical effectuates cyclization onto the neighbouring sp^2^ carbon to form a new C-N bond and an adjacent carbon centered radical. Again, the following oxidation is different for both methodologies. In the electrosynthesis, rearomatization probably occurs through deprotonation and consecutive anodic oxidation to yield the lactam product. In the photoredox catalyzed method, the intermediate radical is intercepted by O_2_ to form a peroxy species. Turn-over of the catalytic cycle and product formation go hand in hand as hydrogen peroxide is eliminated from the product by oxidation with the reduced Ir^II^ catalyst. The advantages of this electrochemical and photocatalytic method are that they use either no oxidant, or otherwise readily available oxygen as an oxidant, respectively.

In both literature reports, phenanthridinones with a wide variety of aryl substituents have been explored. Nonetheless, their scope is very different regarding the type of secondary amide substrates. The Zeng group found that the *N*-acyloxy group is ideal for lactam formation using their electrochemical method. The Hong group, on the other hand, uses *N*-aryl amides in their photoredox catalyzed lactamization. Despite this difference, both demonstrate the ability to synthesize phenanthridinone derivatives bearing a variety of substituents in good yields. Both methods resolve a regioselectivity issue, which is prominent in C-H amination, primarily showcased in the electrochemical method [35]. *Meta*-substituted substrates resulted in C-N bond formation, either exclusively or predominately at the more sterically accessible aryl C-H bond. This way the more preferable 3-substituted lactams were formed. Besides phenanthridinones, both methodologies have a versatile extension to their scope.

With the same photoredox catalyzed method, the synthesis of quinolinone derivatives from *N*-phenylcinnamamides was made possible. Prior art had shown that (*E*)-olefins could be efficiently and selectively converted into (*Z*)-olefins by photoisomerization [36,37]. By means of this method, the excited photocatalyst *Ir^III^ performs a dual role in the reaction. First, it achieves the in situ generation of (*Z*)-*N*-phenylcinnamamides by eletron transfer for *E*/*Z* isomerization. Second, it serves as a photooxidant to generate the amidyl radical in a PCET event. The electrochemical method showed one example of a quinolinone, which was feasible due to its symmetry, circumventing the E-Z isomeric problem. In the electrochemical method, one example of a quinolinone is shown where smart choice of substitution pattern circumvents the *E*/*Z* isomerization requirement. Distinctly, the electrochemical strategy has been shown to tolerate a propenyl and alkyl moiety to afford quinolinone and indolinone products. Even the C(sp^3^)-H amination was made possible given a small modification, in spite of lower yet moderate yields.

As described above, both strategies are quite similar on a mechanistic level. However, the intricate differences result in a different scope, making the two methods complementary. Yet, primary amides are tolerated in neither of the two methods. The free amide has been reported to undergo a Hofmann rearrangement under near-identical electrochemical conditions [38]. Otherwise, the effective BDFE (bond dissociation free energy) for the N-H bond of a primary amide does not match this specific photocatalyst/base pair [39].

Even though both methods are unable to use primary amides as substrates, the advantage of the electrochemical method is that the unsubstituted phenanthridinones can be obtained after deprotection of the *N*-acyloxy group. In case of the *p*-methoxyphenyl substituted amides, the photoredox catalyzed method provides a way to afford unsubstituted phenanthridinones after deprotection of the PMP group. Needless to say, the desired product is a key deciding factor upon the determination of which methodology to use. Within the given conditions, the discussed strategies could seem restricted in their amide scope. The chosen conditions impose an inherent oxidation potential window, which brings forward a set of substrates that can be oxidized within these limits. Of all the examples within this review, this comparison shows best how dialing into the reactivity of the substrate could impact the scope. In electrochemistry this can be done by changing the active potential and/or the redox mediator. Correspondingly, in photoredox catalysis, the photocatalyst could be selected or designed in order to fit the redox potential of the desired substrate [40]. Here specifically, using PCET, the effective BDFE of any given oxidant/base pair can be modified over an arbitrarily wide range of energies.

### 2.3. Intramolecular Oxidative Annulation of N-Aryl Enamines: Indole Synthesis

Indole represents one of the most important scaffolds in bioactive compounds and natural products and is consequently a target of extensive methodology research [41,42,43,44,45,46,47,48,49]. Aside from the Fischer indole syntheses [50,51,52,53,54], plenty of novel modern synthetic approaches exist [42,46,55,56,57]. These approaches typically start from *ortho*-halogenated anilines or alternative complex substrates, which restricts these methodologies in their versatility due to high cost and limited availability of starting materials. One of the most straightforward transformations is the direct C-H activation of *N*-aryl enamines. Synthetically, previously described methods accomplished this by using stoichiometric amounts of external oxidants such as Cu(II) salts [58,59,60], hypervalent iodine reagents [61,62] and *N*-bromosuccinimide [63].

**Figure 3:**
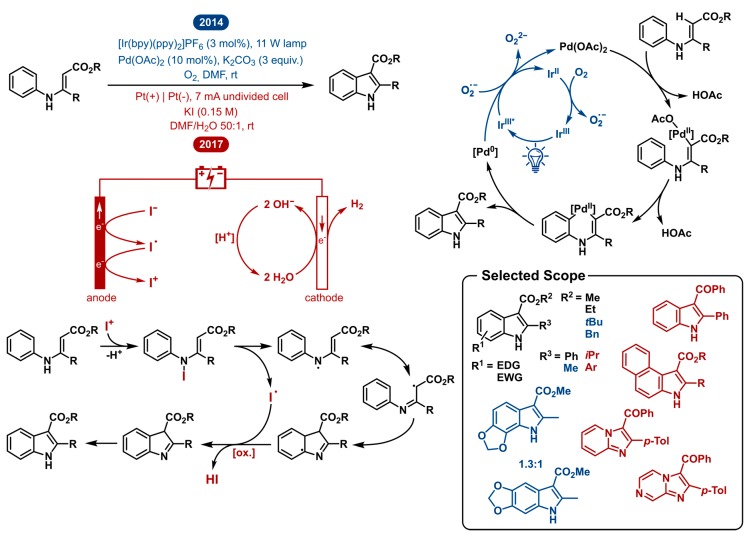
Side by side, comparative visualization of the visible-light-promoted and electrochemical intramolecular oxidative annulation of *N*-aryl enamines regarding reaction conditions, mechanism and product scope.

In 2014, Rueping reported the combined palladium- and photoredox-catalyzed C-H olefination enabling the synthesis of indole derivatives (Figure 3). A catalytic amount of a photoredox active iridium complex in the presence of visible light manages the re-oxidation of the palladium catalyst. In this way, the typical high loadings of external oxidants or metal salts could be avoided. Further control experiments gave interesting mechanistic insights showing that either (a) the iridium catalyst in the absence of oxygen or (b) the in situ formed superoxide anions resulting from regeneration of the photoredox catalyst with oxygen function as the external oxidant. For this reason, this method serves as an alternative to common methods relying on peroxo species. Photoredox catalysis enables to remodel this transformation as a more atom-efficient reaction under mild reaction conditions making it well suitable for syntheses using oxidant-sensitive substrates.

In 2017, the Lei group reported the electrocatalytic reaction protocol for achieving the same direct intramolecular C-H activation of aromatic enamines for the synthesis of indoles. As can be seen in Figure 3, their approach effectively is different. Though starting from the same substrates and obtaining the same reaction products, the mechanistic blueprint is completely different. Where the photoredox approach maintains the established palladium cycle and improves the re-oxidation step, the electrocatalytic approach works under external oxidant-free and transition-metal-free conditions. Mechanistic insights led to a plausible mechanism where after two anodic oxidations, an I^+^ intermediate can be generated from an iodide anion. Next, the *N*-aryl enamine reacts with the in situ generated I^+^ to form an *N*-iodo intermediate. The labile N–I bond homolyzes to reveal the high energy *N*-centered radical and the corresponding iodine radical. This aminyl radical tautomerizes to a lower energy carbon centered radical. This radical can intramolecularly add to initiate the cyclization process. After oxidation and deprotonation, the indole product is formed. This is possible by way of anodic oxidation or the in situ generated iodine radical. Concurrently, cathodic reduction of water produces hydrogen gas. This electrocatalytic approach arguably provides a more environmentally friendly synthesis of indoles as it employs external oxidant-free and transition-metal-free conditions. Although it is tenable that platinum leaching from the electrodes is a possibility, no metal catalysts are used as KI not only acts as the electrolyte but also takes part in the redox processes of the reaction.

Markedly, DMF was crucial in both methodologies as the solvent of choice. For the electrochemical method, water was added as the co-solvent to sustain the cathodic reduction, producing hydrogen gas and hydroxide base. Contrastingly, the photoredox-palladium dual catalytic C-H activation requires excess base in order to achieve good yields.

Both the electrochemical as the photoredox-palladium dual catalyzed system allow the synthesis of highly functionalized indoles. The substrate scope consists mainly of *N*-aryl enamine esters. The dual catalytic strategy tolerates both electron withdrawing as donating groups on the *N*-phenyl ring with slightly lower yields for electron donating groups in the *para* position. The opposite is true for substitutions in the *ortho* position. The tricyclic benzodioxole product was isolated as a mixture of regio-isomers. Additionally, different indole-3-carboxylate ester products were obtained (Me, Et, *t*-Bu, Bn). The electrosynthetic method tolerates various substituents on the *N*-phenyl ring as well as on the phenyl on the 2-position of the indole product. Replacing the C-2 substituent from a phenyl group to the isopropyl group led to decreased reaction efficiency. This could suggest that for the synthesis of 2-arylindoles, the electrochemical method is preferable and for the 2-alkylindole products, the dual catalytic strategy is more suitable in terms of isolated yield. In addition, the electrochemical method still worked upon replacing the carboxylic ester by a ketone, notwithstanding the reaction yield decreased as well. Finally, *N*-pyridyl and *N*-pyrazinyl enamines could be used to afford imidazo [1,2-*a*]-pyridine and imidazo[1,2-*a*]pyrazine products.

Even though both methodologies use two very different modes of activation, the scopes are quite similar. This might come naturally as both make use of the characteristic reactivity of the vinylic C-H bond in the enamine. The latter originates from the two adjacent functional groups, increasing the polarization of this vinylogous bond.

### 2.4. Carbazole Synthesis

The advances observed in carbazole synthesis were very similar to those in indole research discussed above. Carbazoles have also gained great interest in recent years for their frequent appearance as an important motif in bioactive natural products, pharmaceutical agents, electronic materials, including photoconducting polymers and organic optoelectronic materials [64,65,66,67,68,69,70,71,72,73,74,75]. For this reason, many efforts have been made towards the synthesis of carbazole derivatives. The transition-metal-catalyzed intramolecular C-H bond amination of *N*-substituted 2-amidobiaryls has long been the most atom economical method [76,77,78,79,80,81,82,83]. However, some of these transformations use hypervalent iodine as the oxidant via PhI(OAc)_2_/Pd(OAc)_2_, PhI(OAc)_2_/Cu(OTf)_2_.

**Figure 4:**
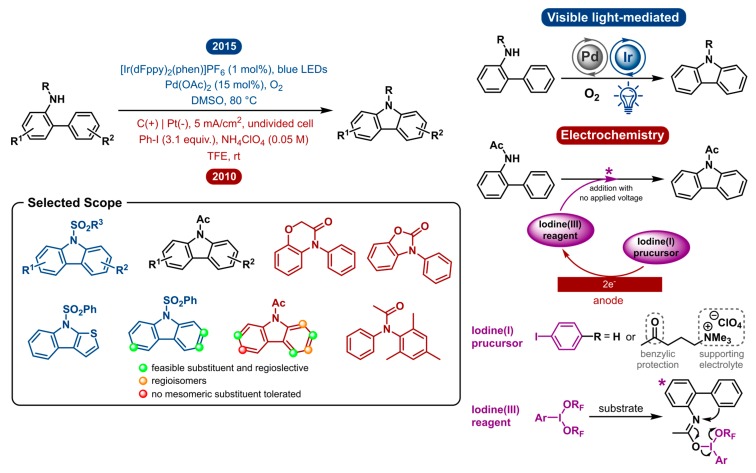
Side by side, comparative visualization of the visible-light-promoted and electrochemical approach to carbazole synthesis regarding reaction conditions, mechanism and product scope. Green = regioselective, orange = non-regioselective isomers, red = no mesomeric substituent tolerated.

In 2010, Nishiyama and co-workers obviated the need for a transition-metal catalyst by way of electrochemically generating stoichiometric amounts of hypervalent iodine, which in turn is used to mediate the C-H amination of diarylamines to yield the carbazole products [84]. The iodine(I) precursor, phenyl iodide (PhI), is electrochemically oxidized in the presence of trifluoroethanol (TFE) to yield the hypervalent iodine(III) reagent PhI(OCH_2_CF_3_)_2_. It possesses comparable or superior properties to commercially available oxidants, such as PIFA [phenyliodine(III)*bis*(trifluoroacetate)] [85,86,87]. Diaryl amines are later added to the hypervalent iodine(III) reagent to perform the reaction. A plausible mechanism (Figure 4) is initiated by S_N_2 attack of the substrate’s amide oxygen to the iodine(III) of the oxidizing reagent to give an imidate-type intermediate. Subsequently, the neighbouring nucleophilic aromatic ring can attack the nitrogen to realize the desired cyclization by C-H amination. This method eliminates the use of transition metals for the synthesis of carbazoles. Nonetheless, it is currently impossible to use substoichiometric amounts of mediator because the exposure of carbazoles to electrochemical conditions results in a complex reaction mixture. The electrolysis of carbazole and its derivatives is quite complex as anodic oxidation of carbazoles leads to homocoupling and electropolymerization [88,89,90]. This exhibits a flaw of this methodology as it uses stoichiometric amounts of chemical oxidants. On the same note, the use of redox mediators often implies an additional obstacle towards product separation. After reaction completion, the redox mediator/oxidant waste must be separated from the reaction mixture. The same counts for the supporting electrolyte, which is idiosyncratic to electrochemistry. Except when the redox mediator and electrolyte are recovered and reused, they are an associated source of waste. Francke et al. aspired to tackle this by developing a redox-active supporting electrolyte by merging an iodine(I/III) mediator with an alkylammonium group [91]. This allowed for the recovery and reuse of both components. The iodine(I) precursor consists of an iodophenyl moiety, in the same way as Nishiyama used PhI, which is attached to a quaternary ammonium group. Both are linked by an alkyl linker that contains a benzylic carbonyl as a protecting group, as the benzylic position is prone to anodic oxidation. The trimethylammonium moiety serves to provide ionic conductivity and is easily separated from the mixture in order to allow recycling of the iodine mediator. This mediator−salt concept was applied to the direct oxidative C−N bond formation, amongst others.

Opposed to the electrochemical strategy, the photoredox catalyzed variation of this transformation is very similar to the indole synthesis discussed in Section 2.3. In 2015, a dual catalytic system was reported by Cho and co-workers, where a Pd-catalyzed method is aided by visible-light-enabled oxidation (Figure 4) [92]. Under an aerobic atmosphere, O_2_ serves as the terminal oxidant to reoxidize the reduced photocatalyst. This merger of palladium and photoredox catalysis circumvents the use of stoichiometric amounts of chemical oxidants or potentially toxic chemical additives. As a result, it is a highly atom economical and environmentally benign version of this transformation.

The visible-light mediated dual catalytic approach has a substrate scope including several *N*-substituted amidobiaryls. The *N*-substitution is of importance as no alkyl groups (Et, Bn) or free aniline could afford the carbamate product. *N*-sulfonyl (benzene-, *p*-toluene- and methylsulfonyl) and *N*-acetyl-substituted amidobiaryl substrates were successfully reacted to produce the corresponding *N*-substituted carbazoles. The reactivity of *N*-acetyl substrates was found to be lower than that of the *N*-benzenesulfonyl analogues. The electronic properties and substitution pattern have no apparent influence on the reactivity. 3′-Substituted substrates could be selectively transformed into single regioisomer products.

The indirect electrochemical method, on the other hand, was heavily influenced by substitution of the two aryl rings and showed different functional group tolerance. Regioselectivity was shown to be a problem for 3′-substituted amidobiaryl substrates which afforded a mixture of regioisomers. This difference could be explained because the photochemical approach goes through the least sterically hindered palladium complex. 2′-Substituted substrates, on the other hand, did afford the desired product and were not included in the scope of Cho and co-workers. Additionally, electron-donating and –withdrawing substituents *para* to the acetamide gave complex mixtures. This was believed to be caused by a resonance effect as a mesomeric *meta* substituent showed no undesired products. A summary of the reactivity is shown in Figure 4. The indirect electrochemical method was also shown to work for the intermolecular C-N bond formation.

In conclusion, both photoredox catalysis and electrochemistry can be used in carbazole synthesis but both perform a very different role in the process. Photoredox catalysis aids palladium catalysis by providing oxidative power but is not directly involved in C-N bond formation. Electrochemistry is also indirectly put to use to pre-electrolyze a (recyclable) mediator, after which the iodine(III) reagent is used for C-N bond formation. Owing to these differences, they show very few similarities in conditions and reactivity.

### 2.5. Decarboxylative Sulfonylation of Cinnamic Acids with Aromatic Sulfonylhydrazides to Vinyl Sulfones

In the last decade, the decarboxylative coupling reaction has emerged as a powerful synthetic strategy that finds great use in the formation of novel carbon-carbon and carbon-heteroatom bonds [93,94,95,96,97,98,99,100,101,102,103,104]. The transformation is very versatile as it starts from inexpensive, easily accessible and highly stable carboxylic acids and typically occurs under mild reaction conditions accessing a broad range of substrates with excellent functional-group tolerance. An example of this is the decarboxylative coupling with sulfonylhydrazides. They make for an interesting coupling partner as they are readily accessible synthetic intermediates that can behave as sulfone sources upon fragmentation by N_2_ elimination. In 2015, Singh et al. reported this decarboxylative sulfonylation [105]. The reaction is I_2_-catalyzed with TBHP as the terminal oxidant starting from aromatic sulfonylhydrazides and cinnamic acids as substrates. In an attempt to reduce catalyst loading, to avoid strong oxidants and to create milder reaction conditions, photoredox- and electrocatalysis gave possible solutions for this challenge.

**Figure 5:**
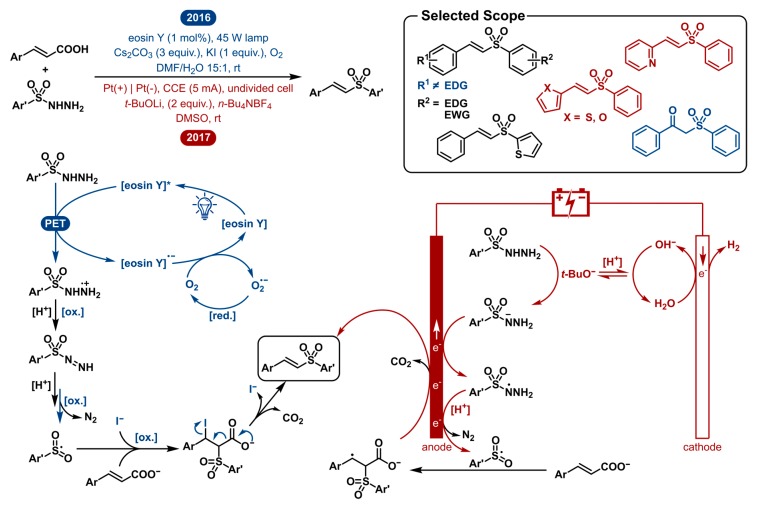
Side by side, comparative visualization of the photoredox catalyzed and electrochemical decarboxylative sulfonylation of cinnamic acids with aromatic sulfonylhydrazides towards vinyl sulfones regarding reaction conditions, mechanism and product scope. (PET: photoinduced electron transfer). EDG = electron-donating group, EWG = electron-withdrawing group.

In 2016, Weng et al. reported the visible-light-enabled decarboxylative cross-coupling of cinnamic acids with sulfonylhydrazides under transition-metal-free conditions [106]. A year later, in 2017, the Huang group disclosed the electrochemical approach for the same transformation [107]. The mechanistic approach is very similar as they both start with the activation of the sulfonyl hydrazide by oxidation (Figure 5). For the photochemical method, the excited eosin Y photocatalyst oxidizes the sulfonyl hydrazide, after which it undergoes sequential N-H abstractions and an oxidation. Oxygen serves as the terminal oxidant by oxidative quenching of the organocatalyst to yield a superoxide radical anion O_2_^•−^, which further oxidizes the intermediate. Subsequent loss of N_2_ yields the key sulfonyl radical. In the electrochemical pathway, deprotonation of the sulfonyl hydrazide followed by quick anodic oxidation and a repeated deprotonation and oxidation yields the same sulfonyl radical after loss of N_2_. This open shell intermediate subsequently reacts with cinnamate. The resulting adduct intermediate is readily decarboxylated and oxidized to afford the vinyl sulfone product. In the photoredox catalyzed method the sequence of radical addition and oxidation is accompanied by an iodide anion I^−^ capturing the adduct intermediate. Elimination of CO_2_ and I^−^ also gives the final sulfone product. Besides a different oxidation approach, both strategies are very similar. By using oxygen as the terminal oxidant or applying electrical current, both methods preclude the need for a strong chemical oxidant. Further, the catalyst loading is low (1 mol%) or absent in the electrochemical case, which is a significant improvement over previous methods. The advantage of the electrochemical method is that it requires neither mediator nor catalyst.

The scope of both procedures is limited by the radical nature of the processes. The key sulfonyl radical intermediate has to exist long enough to react. This limits the scope to aromatic sulfonylhydrazides. In the case of alkyl sulfonyl radicals, loss of SO_2_ will generally generate a more stable sp^3^-carbon derived radical. The instability of phenyl and other aryl radicals prevents this from happening. Furthermore, both scopes are very similar. A series of cinnamic acid derivatives and aryl sulfonylhydrazides were screened. Surprisingly, the α,β-unsaturated carboxylic acid scope showed very different functional group tolerance and reactivity. The photocatalytic method was unable to afford products in reasonable quantities when an electron-donating group (i.e., Ph-OMe or thienyl) was present. Alternatively, methoxy-substituted cinnamic acids present good yields using the electrochemical equivalent. Also, (strongly) electron-withdrawing groups (CF_3_, CN) on the cinnamic acid decrease the electrochemical reaction yield but perform better when employed in the photoredox catalyzed method. Sulfonylhydrazides could be employed with both the electron-donating and electron-withdrawing substituents to furnish the corresponding products in diverse yields with no specific reactivity pattern. Other than that, the photoredox catalyzed transformation was shown to produce the oxygenated β-keto sulfone product while only changing the solvent to DCE in 38% isolated yield. This once more showcases the importance of solvent in these reactions. Though both strategies showed a different preference in the solvent studies, the electrochemical method also worked decently well when DMF was used.

### 2.6. Hydroamination of Olefins

**Figure 6:**
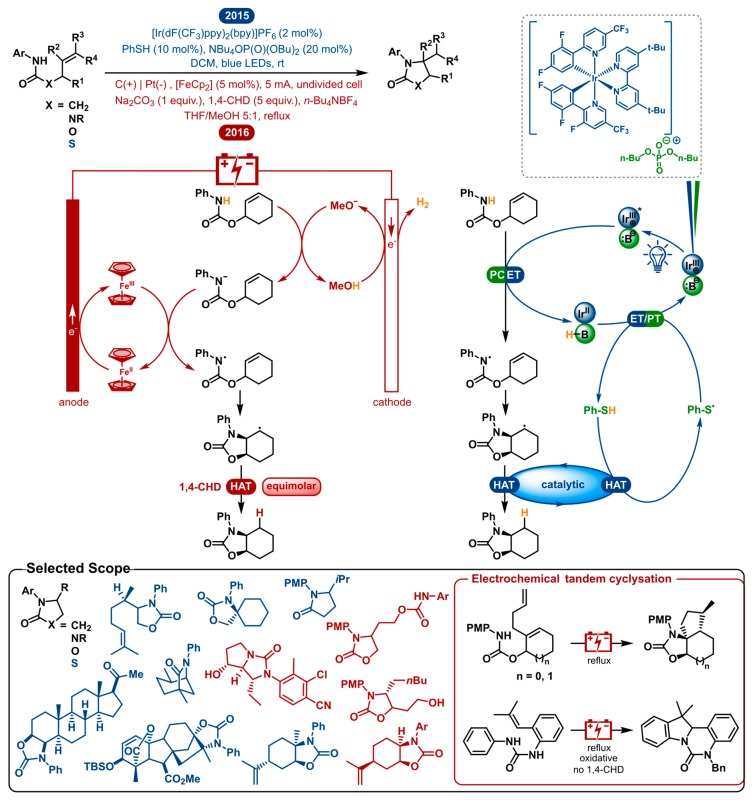
Side by side, comparative visualization of the photoredox catalyzed and electrochemical hydroamination of olefins regarding reaction conditions, mechanism and product scope.

Olefin hydroamination is a very attractive method for C-N bond formation. For this reason, much effort has been put into designing novel synthetic methods for this transformation [108,109,110,111,112,113,114,115,116]. Possibly the most desirable route is the utilization of nitrogen-centered radicals, which are versatile intermediates for the construction of nitrogen-carbon bonds. In 2015, the Knowles and Xu group independently reported a catalytic olefin hydroamidation using amidyl radicals [117,118]. Preliminary work had already shown that methodologies using these open-shell intermediates have the benefit of an extensive scope, consistent anti-Markovnikov regioselectivity and low kinetic barriers moving towards C−N bond formation [119,120,121,122,123,124,125,126]. Although the oxidative generation of these synthetically valuable amidyl radicals is attractive, it still remains a challenging task. In the following section, the photo- and electrocatalytic hydroamination of olefins are discussed (Figure 6).

All previous methods had to rely on uncommon and expensive prefunctionalized substrates or required either stoichiometric or excess amounts of strong oxidants. In this way, catalytic amidyl radical generation using amide substrates under redox-neutral conditions considerably benefits the applicability and atom economy of these transformations. Knowles et al. accomplished this using a triple catalytic system that enables proton-coupled electron transfer (PCET) [117]. An iridium photocatalyst and weak phosphate base cooperatively cause PCET homolysis of the strong anilide N-H bond, generating the amidyl radical. This reactive species then cyclizes onto an adjacent olefin to form a new C-N bond. The following radical intermediate is reduced by thiophenol (PhSH) which acts as a hydrogen atom transfer (HAT) catalyst to yield the product. The PCET and HAT systems cooperatively accomplish each other’s turn-over, closing the dual catalytic cycle. This nicely shows the advantage of this redox neutral method.

Shortly after, in 2016, the Xu group reported the electrocatalytic version of the same transformation [118]. As can be seen in Figure 6, the general layout of the mechanism is parallel to that of the photoredox catalyzed method. The first important difference is that in this electrocatalytic mechanism, a deprotonation is followed by the single-electron oxidation of the conjugate base rather than a concerted PCET activation step. The carbon anode, being the working electrode, oxidizes the redox catalyst, ferrocene (Fc), while the platinum counter electrode conjoins the reaction by reducing MeOH to H_2_ and MeO^−^. After the methoxide base deprotonates the amide, it will be rapidly oxidized to furnish the amidyl radical. The second crucial difference is that a sacrificial HAT reagent is used to reduce the radical intermediate instead of using a HAT catalyst in the photoredox process. This shows that photoredox catalysis is more able to take advantage of the redox-neutral nature of this transformation, as it does not need any terminal oxidants or reductants. Unless an oxidative and a reductive event are coupled, this cannot be realized with electrocatalysis. This strategy is called paired electrolysis and shows comparable utility to redox-neutral and other multicatalytic photoredox chemistry. Nonetheless, it is often hard to pair two half-reactions in a clean manner. For the model reaction, Xu reports 80% isolated product in the absence of 1,4-cyclohexadiene, compared to 90% in its presence. They propose that H-atom abstraction from a solvent molecule is also possible as the final step.

As both strategies are very similar in nature, they afford an analogous scope. A selected scope is highlighted in Figure 6. Besides general amide substrates, ureas and carbamates were also shown to cyclize to the desired products by both methodologies. Moreover, a thiazolidinone product was synthesized from a thiocarbamate by photocatalysis. Both strategies display a multitude of reactions with mono-, di- and trisubstituted olefins. The hydroamination of a tetrasubstituted olefin was also shown to be possible by the visible-light mediated approach. Different substitution patterns on the *N*-aryl group showcasing a wide variety of steric and electronic properties were viable. Included are some oxidation-sensitive functional groups (thioethers, primary alcohols, sulfonamides and competing amides), of which some are generally incompatible with the other modern methods. The two methods experienced no chemoselectivity problems transforming polyolefins. The synthesis of biologically relevant molecules was incorporated in both scopes. The Knowles group performed their hydroamination method on a progesterone- and gibberellic acid derived carbamate yielding high diastereoselectivities and good yields. The Xu group improved the synthesis of an androgen receptor modulator, reducing the number of steps from 7 to 4, including the key hydroamination step. A multitude of cyclic olefins were transformed to provide polycyclic products. Additionally, the electrosynthesis of polycyclic *N*-heterocycles was demonstrated by a radical cascade reaction allowing tandem cyclization of diene substrates or the oxidative addition to the *N*-phenyl group. Both methodologies are restricted to secondary amide derivatives. Nevertheless, oxidative cleavage of *N*-PMP containing products allows for the access to *N*-unsubstituted product classes. Generally good to high diastereomeric ratios are obtained, being comparable between both methods.

### 2.7. Cross-Dehydrogenative Coupling of Tertiary Amines with Ketones

The direct assembly of carbon-carbon (C-C) bonds starting from two simple carbon-hydrogen (C-H) bonds is the ideal scenario for fundamental synthetic transformations. This direct catalytic cross-coupling between two hydrocarbons is termed cross-dehydrogenative coupling (CDC), for which many advances have been reported in the last decade [127,128,129,130,131,132]. An example is the oxidative coupling of tertiary amines with ketones. In 2017, the Luo group reported a photoredox catalyzed as well as electrochemical version of this transformation [133,134]. For this reason, it is an ideal study case for this review.

**Figure 7:**
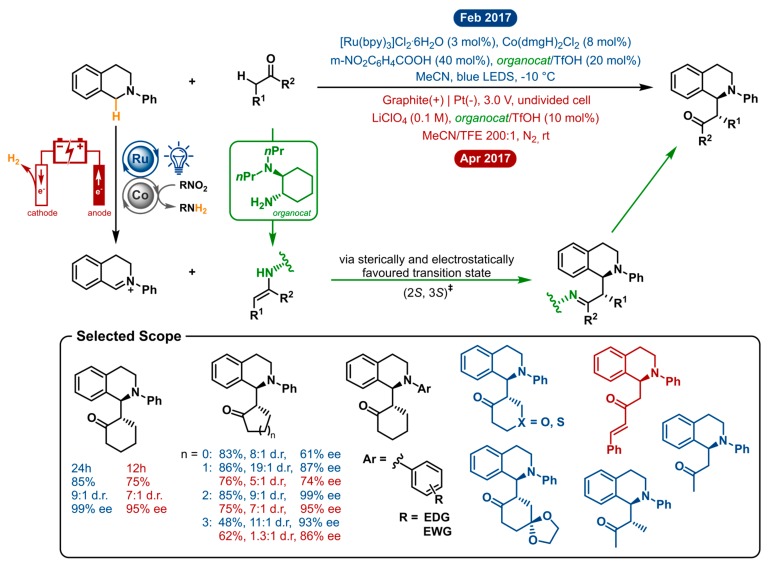
Side by side, comparative visualization of the photoredox catalyzed and electrochemical cross-dehydrogenative coupling of tertiary amines with ketones regarding reaction conditions, mechanism and product scope. *ee* = enantiomeric excess, d.r. = diastereomeric ratio, EDG = electron-donating group, EWG = electron-withdrawing group.

Both methods employ the same general strategy to enable asymmetric cross-dehydrogenative coupling (Figure 7). Organocatalysis is used to gain enantiocontrol over the reaction. In particular, aminocatalysis is operated in synergy with established C-H activation cycles to enable highly stereoselective C-H functionalizations. Chiral primary amine catalysis is coupled with either photoredox-cobalt dual catalysis or electrochemistry to gain access to alkylated tetrahydroisoquinolines. In both cases, the interaction between the chiral amine catalyst and ketone will generate a chiral enamine intermediate. The iminium generation from the tertiary amine substrate is where the difference lies. First off, this is performed by cooperative photoredox Ru/Co catalysis with a substoichiometric amount of nitrobenzoic acid as a hydrogen acceptor (yet, not in terms of redox count). The latter acts as the sacrificial oxidant, obviating the need for excess amounts of strong oxidants and averts oxidative consumption of the organocatalyst. The electrochemical method generates the iminium intermediate by a twofold anodic oxidation. Subsequently, the iminium cation is intercepted by the enamine intermediate through the sterically and electrostatically favoured transition state (2*S*, 3*S*)^‡^. After hydrolysis, the desired product is formed, and the chiral amine can take part in the next catalytic cycle.

The two distinct systems were optimized separately and showed markedly different performance under similar conditions. This can likely be explained by the nature of the oxidation process being so different. Whereas one occurs in a dual catalytic homogeneous fashion, the other oxidation event takes place at an electrode. Both strategies obviate the need for stoichiometric or excess amounts of strong oxidants by using a substoichiometric amount of a nitro-compound acting as the hydrogen acceptor and electricity as a green oxidant, respectively. After iminium ion generation the catalytic enamine intermediate takes stereo control over the reaction. The visible light mediated approach requires decreased temperature (−10 °C) and 20 mol% of organocatalyst to achieve excellent enantioselectivities. Here lies an advantage of the electrochemical method, which can operate at room temperature and only needs 10 mol% of organocatalyst to yield similar—but lower—*ee*’s (vide infra). Acetonitrile proved to be the ideal solvent in both optimizations. For the electrochemical approach, trifluoroethanol (TFE) was found to be a crucial additive. It is proposed to capture the iminium ion intermediate to produce a more stable hemiaminal intermediate, which will further facilitate the C-C bond formation.

By preventing the use of strong chemical oxidants, both methodologies ensure high functional group tolerance and good chemoselectivity. For both strategies, the cross-dehydrogenative coupling was optimized using *N*-phenyl-tetrahydroisoquinoline and cyclohexanone as the model reaction. Here, the numbers, already point out that the photoredox-cobalt-organo triple catalytic method is superior over the electrocatalytic-organo catalytic method in both isolated yield (85% vs. 75%), diastereoselectivity (9:1 d.r. vs. 7:1 d.r.) and enantioselectivity (99% *ee* vs. 95% *ee*). This general trend is also reflected in the rest of the analogous scope. A sequence of cyclic and a few acyclic ketones were shown to form the *syn*-Mannich-type adducts with good yields and high diastereo- and enantioselectivity. Cyclic ketones, from 4- to 7-membered, were shown to afford the products in moderate to high yields (except for cyclobutanone in the electrosynthetic method). However, enantio- and diastereoselectivity were troublesome with the CDC reaction of cyclobutanone and cyclopentanone. Cycloheptanone gave lower yields for both reactions and hardly any diastereoselectivity and reduced *ee* for the electrochemical method. Both methodologies tolerated *N*-aryl-tetrahydroisoquinolines substituted with electron-donating or electron-withdrawing moieties on the *N*-phenylgroup. The visible-light-promoted strategy is not restricted to cyclic ketones, as tetrahydropyranone and tetrahydrothiopyranone were also used as nucleophiles in the CDC reaction. Notwithstanding, the obtained products were found to easily racemize upon isolation by silica gel chromatography. In total, three acyclic ketones were applied in both catalytic systems resulting in good yields but low enantioselectivities (25–74% *ee*). As a conclusion, the photochemical method has the upper hand regarding both the extensiveness of the scope and stereoselectivity. Nonetheless, the electrosynthetic method is oxidant free and less complex, being monocatalytic compared to the triple catalytic process. Here, the direct anodic oxidation as a feature is substrate dependent and requires some optimization for each instance. On the other hand, upon optimization, the electrosynthesis is more conveniently scalable (a 1 mmol scale reaction was performed with no loss of stereoselectivity).

Furthermore, many similar transformations are known in both research fields that use the same approach of iminium ion generation and trapping thereof [1,2,3,135]. Because they could be analyzed analogously to this this section, the summation of these transformations is not included in the scope of this review.

### 2.8. Nitrate Radical Induced Alkyne Oxygenation to 1,2-Diketones and Other Transformations

The nitrate radical (NO_3_^•^) is an inorganic radical which has interesting reactivity towards organic species. Its high reactivity can be ranked between that of the hydroxyl (OH^•^) and sulfate (SO_4_^•−^) radical [136]. Despite the versatility of this reactive species, few applications in organic synthesis have been reported so far. For laboratory use, the only ordinarily used strategies for nitrate radical generation are the reaction of nitrogen dioxide and ozone [137], decomposition of N_2_O_5_ [138,139,140], and the electrooxidation and photoinduced electron transfer of inorganic nitrates or nitric acid [141,142]. The two former strategies are not ideal due to the use of toxic gases. Moreover, the presence of the highly reactive gases will promote an abundance of unwanted side reactions. In 2007, Wille and Andropof demonstrated the use of the nitrate radical for the oxygenation of alkynes [143]. The nitrate radicals were both electro- and photochemically generated and compared as viable techniques. For the latter, ceric(IV)ammonium nitrate (CAN) is used, which under irradiation with UV-light undergoes photo-induced electron transfer. The electrochemical strategy directly generates the nitrate radicals from nitrate anions via anodic oxidation.

**Figure 8:**
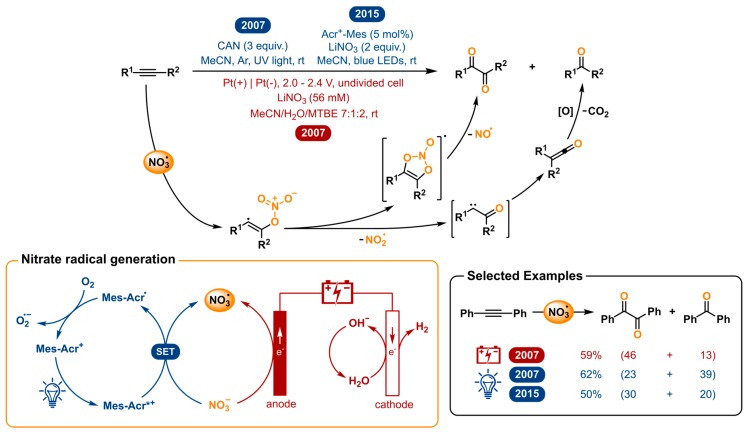
Comparative visualization of the photochemical and electrochemical oxygenation of alkynes towards 1,2-diketones regarding reaction conditions, mechanism and product scope.

When NO_3_^•^ was reacted with aromatic alkynes, 1,2-diketones were formed as the major product (Figure 8). Surprisingly, considerable amounts of the benzophenone were also detected. After experimental and computational investigation, the following mechanism was proposed to explain both products; in spite of a difference in nitrate radical generation, the mechanistic path followed by the substrate to give both products is the same for both methods. The addition of NO_3_^•^ to diarylacetylenes leads to a nitrate adduct vinyl radical. This highly reactive and unstable species finds one of two ways to react. It can undergo 5-*endo* cyclization to the five-membered intermediate, which in its turn undergoes the loss of nitrogen oxide NO^•^ to yield the 1,2-diketone product. Alternatively, the vinyl radical can lose a nitrite radical NO_2_^•^ via γ-fragmentation. Wolff-rearrangement of the resulting α-oxo carbene to the ketene and subsequent oxidation and decarboxylation gives the benzophenone product. Photogeneration of NO_3_^•^ using CAN was found to result in poorer 1,2-diketone/benzophenone ratios, compared to the electrochemical method. They suggest that the aromatic diketones absorb light at the same wavelength as the NO_3_^•^ precursor CAN and could therefore photochemically decompose. For this reason, electrochemical generation of nitrate radicals was concluded to be the preferable method.

A few years later, Wille collaborated with the König group to develop a novel methodology for the photochemical nitrate radical generation [144]. The revival of photoredox catalysis gave an opportunity to revise this transformation and make the need for excess of CAN and UV light unnecessary. As the photocatalyst, 9-mesityl-10-methylacridinium (Mes-Acr^+^) was chosen for its strong oxidizing power in the excited state [145]. After irradiation by blue light, the excited photocatalyst is capable of oxidizing the nitrate anion to elegantly generate the nitrate radical. In this way, not only the stoichiometric excess of cerium reagent and the use of UV light is replaced by a catalytic amount of organocatalyst and lower energy blue LEDs, but also the NO_3_^•^ can be generated from simple inorganic nitrate salts such as LiNO_3_. It was demonstrated that this method could oxygenate alkynes with improved yields and selectivity towards the desired 1,2-diketone product over the former photochemical method. Nonetheless, the electrochemical method has a better 1,2-diketone/benzophenone ratio. The nitrate radical has also proven to be useful in other work from Wille and co-workers for the self-terminating radical oxidative cyclization to tetrasubstituted tetrahydrofurans using either CAN photolysis or electrochemical oxidation of LiNO_3_ [146,147] and was repeated with the photoredox catalyzed version [144].

In conclusion, this section nicely shows how photoredox catalysis and electrochemistry are able to generate the same radical species, which in turn performs a specific transformation, yet side reactions innate to the approach make one preferable over the other. In the present case, photodegradation of the product highlights a flaw of photoredox catalysis and photochemistry in general.

### 2.9. Vicinal Difunctionalization of Alkenes: Chlorotrifluoromethylation

Vicinal difunctionalization of alkenes is an effective and valuable method to introduce two new adjacent functional groups [148,149,150,151,152]. Stereocontrol can often be exerted, particularly if both bonds are formed simultaneously or when activated double bonds show distinct philicity towards nucleophiles or electrophiles [153]. This makes this strategy highly reliable. In this section we will discuss the chlorotrifluoromethylation enabled by both photoredox catalysis and electrocatalysis.

In 2014, the Han group reported the photoredox-catalyzed vicinal chlorotrifluoromethylation of alkenes using a ruthenium photocatalyst [Ru(Phen)_3_Cl_2_] [153]. CF_3_SO_2_Cl is used as the reagent, which is activated by the excited photocatalyst, so both the CF_3_ radical as the chlorine source Cl^−^ are discharged, releasing SO_2_ as the only byproduct. The electrophilic CF_3_^•^ reacts with the electron rich-alkene substrate giving the radical intermediate. The [Ru(Phen)_3_]^3+^ catalyst is regenerated by oxidizing said intermediate into a carbocation, which in turn is trapped by Cl^−^ to yield the product. This redox neutral process has the advantage of not requiring an external oxidant or reductant. It is accompanied by the disadvantage of needing CF_3_SO_2_Cl which is highly reactive and delicate to handle, which could be problematic on a bigger scale (bp: 29–32 °C).

**Figure 9:**
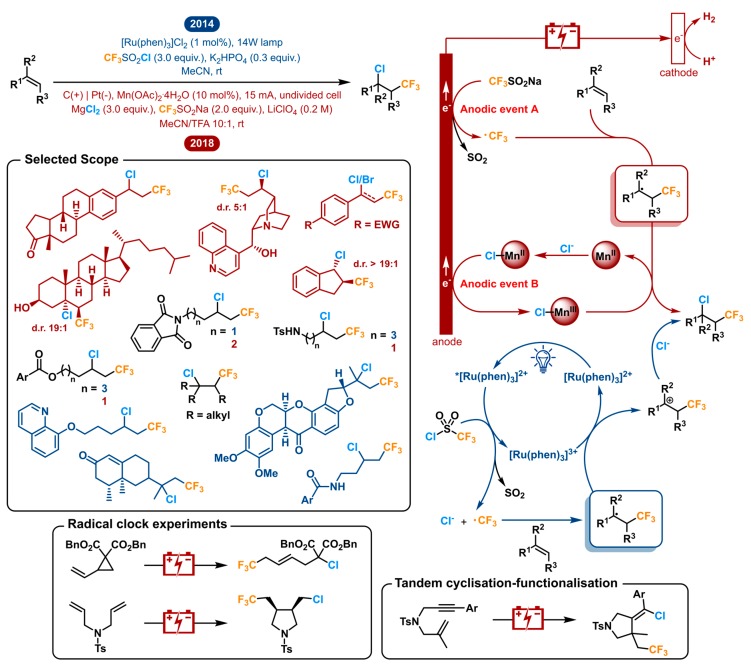
Side by side, comparative visualization of the photoredox catalyzed and electrochemical cross-dehydrogenative coupling of tertiary amines with ketones regarding reaction conditions, mechanism and product scope.

In 2018, the Lin group reported the electrochemical approach to the chlorotrifluoromethylation of alkenes [154]. They opted to use the Langlois reagent (CF_3_SO_2_Na) as their trifluoromethyl source, combined with MgCl_2_ as the chloride source. Both are attractive solids to use because they are commercially available and bench-stable. The challenge here is to get both to react in a predictable manner. Where the photochemical strategy started off by reducing its CF_3_ source, the Langlois reagent has to be oxidized in order to generate CF_3_^•^. The consecutive electrophilic addition to the alkene is the same, giving the corresponding radical intermediate. Wanting a chloride anion Cl^−^ to react with said intermediate, one has to oxidize either of both. For this reason, Lin used the concept of parallel anodic oxidation (or anodically coupled electrolysis), where a completely separate, additional anodic event B takes place (Figure 9). In addition to MgCl_2_, a catalytic amount of Mn(OAc)_2_ is used which is anodically oxidized to generate [Mn^III^]-Cl. It reacts with the intermediate radical as the chlorinating agent. In this way chemo- and stereoselectivity can be achieved. The transient CF_3_ radical adds faster than the persistent metal-based transient radical ([Mn^III^]-Cl) after which the radical cross-coupling of a transient and a persistent radical outcompetes that of two transient carbon-centered radicals. For this reason, the heterodifunctionalization is favoured in the stereoselectivity in which it is observed. The two independent optimizations both resulted in acetonitrile being the favoured solvent.

In spite of the fact that both reports opt for a very different approach, they are able to generate a very similar scope. A selected scope is shown in Figure 9. The molecules displayed in black illustrate the lapped substrates between both reports, which include some terminal alkenes with ester, amide and amine functionalities. Both strategies were also able to afford the desired products from 1,2-disubstituted and trisubstituted internal alkenes. The Lin group also reported the electrochemical difunctionalization of a tetrasubstituted system. In addition, they demonstrated that, in their hands, this transformation only afforded a small amount of product using the photochemical method of the Lin group. The same exercise was done on a few other substrates such as a styrene and phenylacetylene among others, with the same conclusion. Other notable examples from the electrochemical scope are cyclic alkenes, being converted to the trans products in high diastereoselectivities, and several natural products (or analogues) in which with secondary alcohol, sensitive tertiary amine and quinoline moieties subsist. The photochemical method also reports the difunctionalization of some biologically active compounds such as rotenone and (+)-nootkatone showing good functional group tolerance. Further, in these conditions the quinoline moiety was shown to withstand CF_3_ addition to the aromatic ring [155]. Additionally, radical clock experiments showed interesting ring rupture and cyclization adducts from vinyl cyclopropane and bisallylamine substrates, respectively. An allylpropargylamine also underwent tandem cyclization-functionalization. Finally, the photochemical method has shown ability to be extendable to bromotrifluoromethylation.

For this segment, these two methodologies were chosen because they were the first of their kind within both fields. Though, in 2015, Dolbier et al. and Reiser et al. reported similar vicinal photoredox mediated chlorotrifluoromethylations. The former uses Cu(dap)_2_Cl as a photocatalyst and focused on chlorotrifluoromethylations of α,β-unsaturated amides and esters (and other EWG’s) [156]. Additionally, a variety of other fluoroalkylsulfonyl chlorides were employed. The latter also uses Cu(dap)_2_Cl as the photocatalyst of choice and demonstrates it as a unique catalyst for the trifluoromethylchlorosulfonylation of unactivated alkenes because it suppresses SO_2_ extrusion [157]. Net addition of CF_3_SO_2_Cl instead of CF_3_Cl provides the formation of a different product class. These other two photoredox catalyzed approaches complement the scope of the initial photochemical report significantly.

This discussion shows how a transformation can be operated by two different toolboxes applying two completely different strategies to difunctionalize olefins, yielding a very similar scope. The photoredox strategy couples two redox events to total a redox neutral series of events. On the other hand, the electrocatalytic method makes strategic use of anodically coupled electrolysis by merging two distinct oxidative events.

### 2.10. Oxygenation of Unactivated C-H Bonds

**Figure 10:**
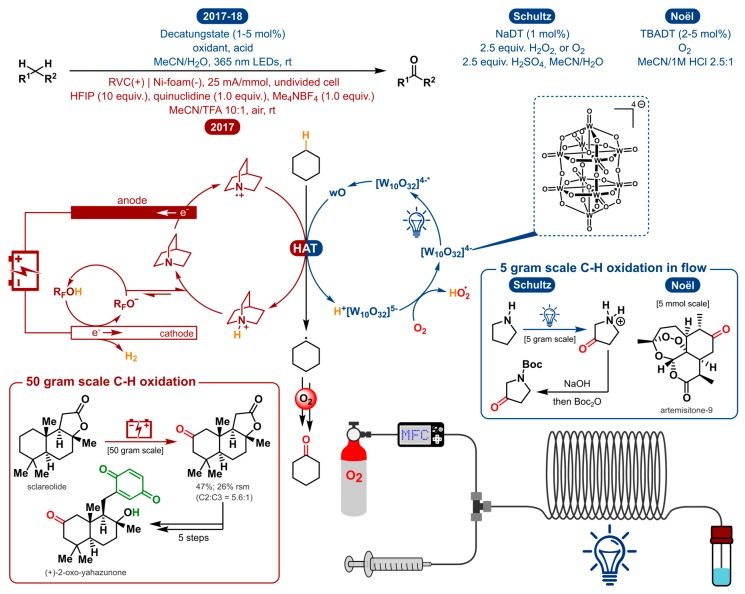
Side by side, comparative visualization of the photochemical and electrochemical oxygenation of unactivated C-H bonds regarding reaction conditions and mechanism. R_F_OH represents HFIP (hexafluoroisopropanol) in the mechanism.

Functionalization of unactivated carbon-hydrogen bonds is one of the more challenging tasks in organic synthesis. In polar chemical conditions, these unactivated C-H bonds are extremely inert making them disinclined to engage in proton or hydride transfer. Rather than overcoming the high acid dissociation constants (pKa), the homolysis of the C-H bond has been the preferred method in a lot of research regarding this topic. Nonetheless, it is still demanding to achieve enough oxidizing power to oxidatively cleave bonds with bond dissociation energies (BDE) this high. At the same time, the oxidation must be selective in order for it to occur prior to the degradation of other moieties. Typically, catalytic systems using various transition metal catalysts were employed to accomplish such transformations [158,159,160,161]. The need for high temperatures, strong and complex oxidants impose scope limitations and impede general adoption of these methods. Hydrogen-atom transfer (HAT) makes for an ideal way to activate C-H bonds. In the 1990′s, Hill et al. introduced the possibility of activating nonactivated C-H bonds using a powerful UV-mediated polyoxometalate HAT catalyst, namely decatungstate (DT) [W_10_O_32_]^4−^ [162]. Others later described possibility for DT to photocatalyze the oxygenation of cyclohexane [163,164]. The following comparative segment discusses the oxygenation of C-H bonds via photo- and electrocatalytic methods (Figure 10).

In 2017 and 2018, Schultz et al. and the Noël group independently reported the first synthetically useful methodologies for the selective C(sp^3^)-H oxygenation using decatungstate photocatalysis (Figure 10) [165,166]. The main differences are the use of a different DT salt (sodium vs. tetrabutyl-ammonium), another general oxidant (H_2_O_2_ vs. O_2_) and acid used (H_2_SO_4_ vs. HCl). But most importantly, the former focuses on the remote functionalization of aliphatic amines and the latter provides a general method for the C-H oxidation of both activated and unactivated aliphatic bonds. In order to access remote C(sp^3^)-H sites within unprotected amines, prior art for the deactivation of the amine α-C-H bonds was used. By protonation of the amine, the less activated, remote C-H bonds become susceptible to oxidation [167]. To get excited, and ultimately accumulate enough energy to cleave a strong C-H bond, decatungstate has to be irradiated by UV light. The initial excited state is produced by oxygen to tungsten charge transfer and decays fast to form the longer-lived species designated as wO [168,169]. The latter is the reactive catalytic species that performs HAT with the substrate, generating a carbon-centered radical. In reaction with oxygen via several steps, most probably involving a peroxo species, the ketone product is formed. Reoxidation of the reduced intermediate will regenerate the catalyst. To achieve the same carbon-centered radical via direct electrolysis, other functionalities, solvents and additives are expected to be oxidized prior to the desired C-H bond.

**Figure 11:**
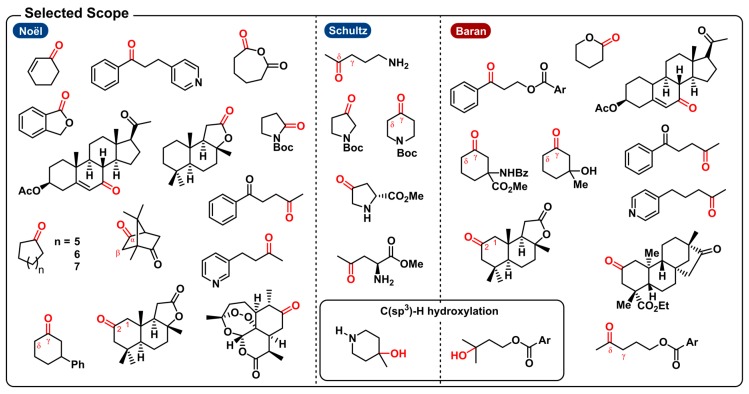
Selected examples from the scope of the three discussed methodology reports discussed in this section. For further details, examine the respective articles.

In 2017, the Baran group tackled this problem by using quinuclidine as a redox mediator in the electrosynthesis to circumvent said degradation [170]. The mediator will be anodically oxidized, generating a reactive radical cation which could facilitate substrate oxidation through efficient homogeneous electron transfer rather than a heterogeneous process occurring at the anode [171]. Moreover, the quinuclidine mediator allows electrochemical oxidation to take place at a relatively low potential. The key HAT activation step is operated by the anodically generated quinuclidine radical cation, in analogy with the excited decatungstate photocatalyst. Consequently, a range of functional groups are tolerated under the given reaction conditions. Figure 11 communicates a selected scope for the three methodologies discussed above.

The photochemical method from the Noël group describes the selective aerobic oxidation of both activated and unactivated C(sp^3^)-H bonds. Examples of activated scaffolds include allylic and benzylic positions and α-alkoxy C-H bonds. Yet, a Boc-protected pyrrolidine was shown to undergo selective oxidation at the α-C-H bond in a Shono-type fashion to afford the protected 2-pyrrolidone in moderate yield (23%) (a similar electrochemical Shono-type oxidation was recently disclosed [172]). Notably, the photochemical method from Schultz et al. is able to produce the protected 3-pyrrolidone from pyrrolidine because the protonation of the free amine makes the aforementioned Shono-type oxidation impossible. Amino acids were also suitable substrates towards oxygenation. (*S*)-proline methyl ester, for example, undergoes oxygenation exclusively at the C4-position. This schowcases some unique regioselectivity for these specific distal (sp^3^)-H bonds. Other substrates, on the other hand, produced a mixture of carbonyl products. For instance, oxygenation of piperidine gave a 2:1 mixture of the δ/γ ketone products. They also noticed that the presence of a remote tertiary C(sp^3^)-H bond leads to hydroxylation of that bond. Likewise, the quinuclidine-mediated approach can also be used for the electrosynthesis of tertiary alcohols in the presence of tertiary C(sp^3^)-H bonds. Regarding the scope of activated C-H bond oxygenations, the electrochemical method is capable of tackling similar allylic and benzylic positions and α-alkoxy C-H bonds. With unactivated aliphatic systems, both the photo- and electrochemical methods induce oxidation of the methylene units most distal to electron-withdrawing functionalities. Moreover, here, indistinct selectivity for the methylene units could lead to a mixture of ketone products. Both approaches will selectively oxidize distal C-H bonds in substrates where the benzylic position is either unavailable or deactivated (e.g., pyridine). Other examples include bioactive compounds such as (1*R*)-(+)-camphor, (+)-sclareolide, artemisinin and an isosteviol derivative.

The scalability of the electrochemical method was shown by the oxygenation of sclareolide on a 50 g scale and incorporated to in the synthesis of (+)-2-oxo-yahazunone. Though the scalability of photoredox catalysis is often questioned, the two photochemical strategies also demonstrated to be scalable with a 5 g and 5 mmol scale synthesis by way of a continuous-flow setup.

As a conclusion, electrochemistry and photoredox catalysis compare very well to each other in regard to this transformation, as they both employ a similar mode of activation (through HAT) and share analogous selectivity, which is not the case comparing it to other C-H oxygenations using metal complexes or strong oxidants (e.g., TFDO).

### 2.11. Photoelectrochemical Chemical Oxidant-Free Minisci-Type C-H Alkylation of Heterocycles

From the previous examples it becomes clear how photoredox catalysis excels in redox-neutral transformations. The nature of the catalytic cycle provides the system with an oxidant as well as a reductant with each turnover of the photocatalyst. On the other hand, net reductive and net oxidative transformations can be performed under electrochemical conditions without the need of a chemical reductant or oxidant as it is substituted by electrical power. Nevertheless, the generated radicals can undergo undesirable over-reduction and over-oxidation, radical homocoupling and electrode passivation. The transient and more diffuse nature of redox mediators, excited photocatalysts and radical species preclude these phenomena. The combination of photoredox catalysis and electrochemistry could lead to a constructive merger filling each other’s flaws by their respective advantages.

**Figure 12:**
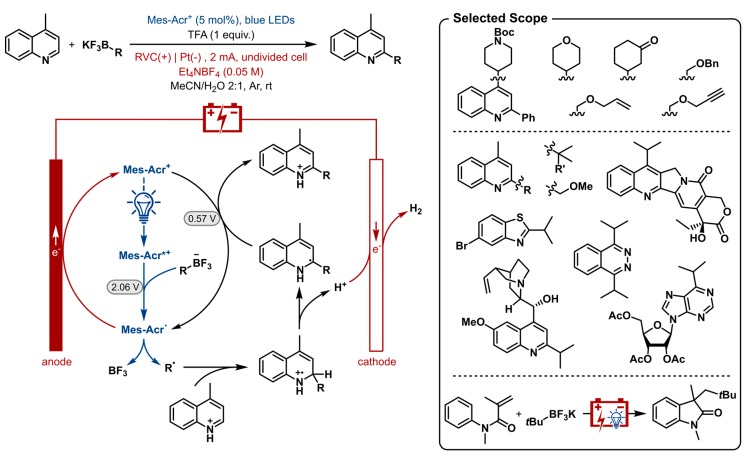
Photoelectrochemical chemical oxidant-free Minisci-type C-H alkylation of heterocycles including mechanism and selected examples.

Recently, Xu and co-workers demonstrated this concept by merging photoredox catalysis with electrocatalysis to enable a chemical oxidant-free Minisci-type C-H alkylation of heterocycles with organotrifluoroborates as shown in Figure 12 [173]. As suggested, the photocatalyst is mechanistically involved in three processes. After irradiation, the trifluoroborate undergoes single electron oxidation by way of the excited photocatalyst (E^red^ = 2.06 V vs. SCE) [174]. After Minisci-type radical addition the heterocycle is reoxidized by the mild ground-state catalyst Mes-Acr^+^ (E^red^ = 0.57 V vs. SCE) [174] to afford the product. Both oxidation processes produce the stable acridinyl radical Mes-Acr^•^, which is in turn anodically oxidized regenerating the ground-state photocatalyst. Concurrent cathodic reduction of protons generates hydrogen gas.

A wide range of aromatic heterocycles were shown in this nucleophilic radical substitution reaction. Several bioactive substrates were included, such as camptothecin, quinine and purine producing a monoalkylated product with good chemo- and regioselectivity. The organotrifluoroborate scope included a variety of cyclic and acyclic primary, secondary and tertiary trifluoroborates. The corresponding radicals include tertiary and α-alkoxyl radicals which have oxidation potentials much lower than their representative organotrifluoroborates and are thereby susceptible to overoxidation to carbocations. Due to this merger of the two catalyst systems and altered conditions this problem was overcome. The photoelectrosynthesis of oxindole was shown as a proof of concept for another chemical oxidant-free transformation by oxidative tandem addition-cyclization of acrylamide.

## 3. Discussion

### 3.1. Underlying Physicochemical Structure and Mode of Activation

**Figure 13:**
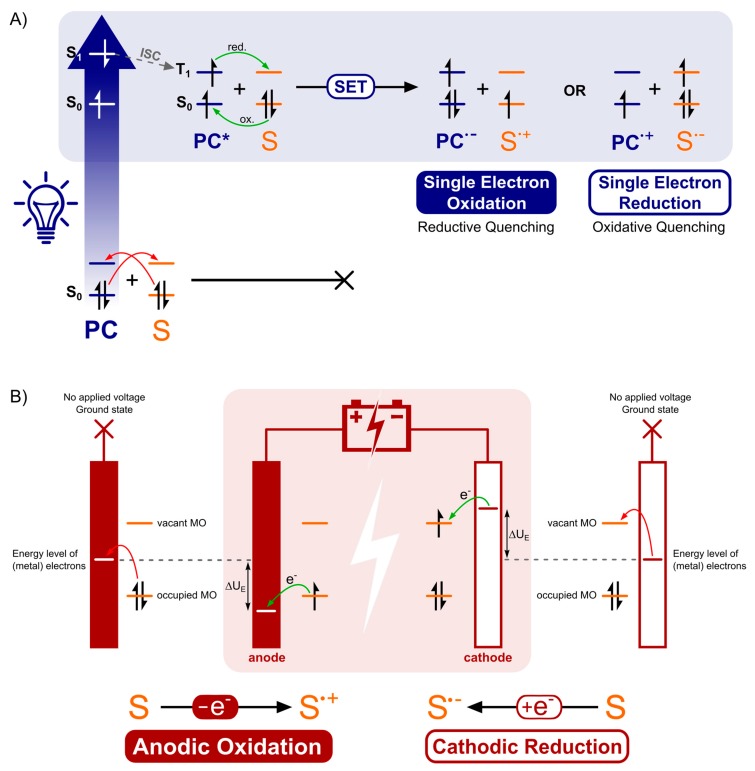
Simplified MO energy diagrams. (**A**) Photophysical processes occurring in the photoredox catalyzed PET activation of substrate molecules (S) by a photocatalyst (PC). (**B**) Depiction of the electrochemical activation of substrates (S) by ET. Red = non-favorable ET, green = favorable ET.

The mechanistic processes and the players in it, examined in Section 2, make it apparent that photoredox catalysis and electrochemistry share a principal mode of action. This being, the activation of substrates by single electron transfer (SET) by assistance of an alternative energy source. In Figure 13, this is visually depicted by the corresponding simplified energy state diagrams. When no external energy source is allowed, the molecular orbitals of a ground state photocatalyst PC and energy levels of the electrodes are positioned unfavorable towards oxidation and reduction of a hypothetical substrate S. The following paragraph is intended to provide a conceptual understanding and does not include an exhaustive discussion of the respective processes. Further, exceptions to these general descriptions exist, including chain mechanisms [175] and indirect electrolysis using mediators [171], but these representations are descriptive for the majority of photoredox catalyzed and electrochemical transformations. For an excellent discussion on these concepts, the following literature exists for both photoredox catalysis [1,2,176] and electrochemistry [177,178].

As depicted in Figure 13A, the external energy input via irradiation will excite a ground state electron in the photocatalyst PC (S_0_) so that it is promoted to a higher energy level (S_1_). The chromophore could return directly to its original ground state (S_0_) by either nonradiative or radiative decay. Yet, a good photocatalyst is defined by its ability to undergo spin forbidden intersystem crossing (ISC, S_0_ → T_1_) to afford an excited, long-lived, triplet state photocatalyst PC* (T_1_). The molecular orbitals of the excited photocatalyst PC* are now positioned thermodynamically favorable towards both single electron oxidation and reduction of a hypothetical substrate S. Both the loss and acceptance of an electron are now beneficial compared to the situation before irradiation (depicted in Figure 13A, by green and red arrows). Quenching of the excited photocatalyst PC* by electron donation to substrate S or an oxidant is called oxidative quenching. Reductive quenching occurs upon quenching of PC* by acceptance of an electron from S or a reductant. The overall process of consecutive photoexcitation of a photoredox catalyst PC and electron transfer with a substrate in the ground state, is called photoinduced electron transfer, or PET.

The mode of activation that characterizes electrosynthesis can easily be compared to that of photoredox catalysis, described above. Just like the unfavorable positioning of molecular orbitals of the substrate S with respect to those of the ground state photocatalyst, the energy levels of the electrode metal electrons without an applied voltage are also inopportunely positioned (Figure 13B, red arrows). Upon application of an external energy source, an electric potential (U_E_), the energy levels of the cathode metal electrons are raised while the anode metal electrons are simultaneously lowered in energy (the same applies to non-metal electrodes, e.g., graphite). At this point, the cathode and anode bear a negative and positive potential, respectively. While driving the potential, the energy level of anode electrons will decrease to such a degree that the HOMO electrons from a hypothetical substrate S in solution will relocate to a more favorable energy level on the anode via an electron transfer process (Figure 13B, green arrow). This is called anodic oxidation of the substrate and brings forth an oxidation current from the solution to the electrode. Alternatively, while the potential at the cathode decreases, the energy levels of the electrode electrons will elevate to a point where they can transfer into the LUMO’s of substrate molecules. This process is termed cathodic reduction, generating a reduction current from electrode to solution. To conclude, external energy input via irradiation and applied voltage drive the activation, by SET of substrate molecules (oxidation as well as reduction) for photoredox catalysis and electrochemistry, respectively.

For both electrosynthesis and photoredox catalyzed reactions, transport of molecules towards and away from their respective “areas of activation” is an important consideration [177,179,180]. For reactions to occur, substrate molecules need to be activated. For electrosynthesis, mass transfer from the bulk solution towards the electrode surface is of fundamental importance. As discussed in Section 3.2, the penetration depth of light is limited, resulting in an analogous scenario where substrate molecules must enter the reactive film in which photochemical activation takes place. As a result, mass transfer from the bulk solution towards these respective areas of activation could limit the rate of the reaction. Diffusion and convection are two basic mechanisms of mass transport. Diffusion is the net transport of molecules from a region of higher concentration to regions of lower concentration. Convection causes the movement of molecules through hydrodynamic stirring. In electrosynthesis, migration, the movement of charged molecules in the electric field is also an important mechanism that contributes to mass transfer. When transport of molecules towards their respective areas of activation leading to the reaction is fast, this leaves the electron transfer reaction as the rate limiting factor. In an ideal scenario the mass transport is high so that it provides substrate molecules fast enough to be activated and react. Additionally, the fast evacuation of product molecules away from the electrode surface or heterogeneous photocatalyst into the bulk of the solution will minimize side reactions (overoxidation, overreduction, etc.). Under the same consideration, redox mediators (indirect electrolysis) and homogeneous photocatalysts are sometimes preferred over their heterogeneous counterparts (vide infra).

### 3.2. Tunability, Advantages and Restrictions

When the mode of electrochemical activation is understood, it is easy to understand that electrochemistry is very tunable. For a given transformation, the redox potentials of various substrates can vary significantly. Yet, for many syntheses, the oxidative or reductive power is statically fixed (dependent on oxidant/reductant) after optimization of reaction conditions. Consequently, the reaction times and/or yields can vary significantly over a set of different substrates. Some substrates will simply be ruled out because the redox activation is thermodynamically impossible. An electrochemical set-up is very tunable, as a potentiostat essentially allows to minutiously regulate the active potential. This enables electrosynthesis by dialing in the redox potential needed for a particular substrate. Because of this, potentiostatic control permits the functionalization of complex synthetic intermediates with great chemoselectivity.

To a similar extent, the tunability in photoredox catalysis is also possible. Recently, significant effort has been spent to tune the electrochemical and photophysical properties of photocatalysts [40,176,181,182,183,184,185,186,187,188,189,190]. Different substitution patterns and ligand usage may lead to different excitation maxima, redox potentials and improved excited-state (and other redox) lifetimes, chemical stability and solubility. As a result of this, the plethora of photocatalysts that are now available provide a means of dialing into the redox potential of substrates to a certain extent. Obviously, the ease of, and precision in which this is possible is still greater with potentiostatic electrochemistry. Cyclic voltammetry provides a quantitative electrochemical analysis as a powerful instrument for investigating the electron transfer processes in reactions [191,192]. Nonetheless, the tunability is not unconditional as it is restricted by “redox potential windows”. In the field of electrochemistry, redox potential windows are termed electrochemical windows and are defined as the difference between the cathodic and anodic limits. These are the potentials at which reduction and oxidation of a given component takes place, respectively [179]. In photoredox catalysis, this window is defined as the reduction potential window attainable by the excited photocatalyst PC* and its oxidized/reduced counterpart (PC^•+^ and PC^•−^). Since both systems are more complex in terms of redox considerations, other boundaries have to be taken into consideration. A common restrictive potential window for both photoredox catalysis and electrochemistry (and other redox chemistry for that matter) is the solvent window [193,194]. An alternate solvent choice can broaden the permissible potential window and/or lower the potential of the desired species, while raising others [118]. For this reason, it is not surprising that for most transformations discussed in this review, the same solvent systems proved to perform best for both methodological approaches. In the case of electrosynthesis, the electrolyte selection also sets an anodic and cathodic limit where both the cation and the anion must be considered. Finally, electrode selection is crucial because they all have their own window of operation, and at a certain point will become sacrificial and dissolve. Broad potential windows are found with platinum, gold and boron-doped diamond (BDD) electrodes. These concepts are useful to discuss separately, but it is finally the interplay of the electrolyte solutions and electrodes that defines the specific electrochemical window. In general, electrochemistry has the advantage of having a broader accessible potential window, as photocatalysts have a confined achievable potential range.

As discussed in Section 2.10, direct electrolysis of substrates can produce some problems. These are often circumvented by using a mediator [171]. As a result, the electron transfer process changes from a heterogeneous process taking place at the electrode (direct electrolysis) towards a homogeneous electron transfer process. This redox catalyst, in analogy with a photoredox catalyst, is an oxidant or reductant by proxy and ultimately also introduces a redox potential boundary. This is not necessarily a disadvantage as it can increase the selectivity of the desired transformation (Section 2.10). Where electrode deactivation by passivation is an inherent problem associated with electrochemistry, photobleaching of the catalyst can similarly pose a significant problem with photoredox catalysis [144,195,196]. One of the factors that help minimize this is the use of light emitting diodes (LEDs). LEDs allow narrow wavelength selection to effectively dial into the absorption spectrum of the photocatalyst towards selective excitation, maximizing energy-efficiency. Nonetheless, the series of wavelength-chromophore couples is still incremental compared to the continuous control over the current and voltage via electrochemistry. Additionally, the attainable redox potential window is inseparable from the wavelength of the light source, which exposes another boundary. UV LEDs are available, yet currently still expensive. Moreover, non-redox photochemical side reactions become more prevalent at shorter wavelengths. This can make it challenging to use certain substrate classes in photoredox catalysis. Problematic compounds include photo-active compounds, which decrease the efficiency of the system by competitive absorption of the light (Section 2.8), undergoing photocycloadditions, bond homolysis or rearrangements. At the same time, a welcome advantage could be the autocatalytic effect of a photoactive product as a (co-)catalyst [197].

In a similar way, some transformations currently seem very challenging via an electrochemical approach. Examples are the usage of carbazoles due to electropolymerization (Section 2.4) [84,88,89,90,91] and the difficulty of employing aldehydes as acyl radical precursors [3] compared to the photochemical approach [198].

**Figure 14:**
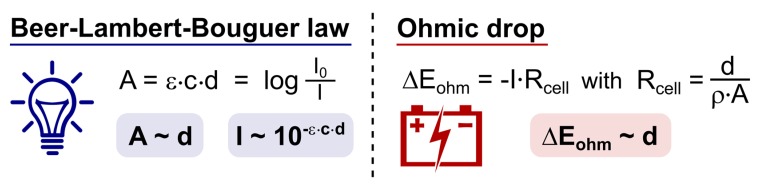
Influence of “distance” (d) on two fundamental physical properties regarding photoredox catalysis and electrochemistry. The light absorption (A) and ohmic drop (ΔE_ohm_) are both proportional to the pathlength of light propagation (d) and interelectrode distance (d). ε = molar extinction coefficient, c = molar concentration, I_0_ = incident intensity, I = transmitted intensity (left), I = electric current (right), R_cell_ = electrical resistance of the cell, ρ = solution specific conductance, A = area of working electrode.

As discussed above, potentiostatic control allows tunability in electrochemical transformations. Constant potential electrolysis (CPE) generally affords better chemoselectivity than constant current electrolysis (CCE, galvanostatic). However, potentiostatic set-ups require a reference electrode (e.g., SCE = saturated calomel electrode or Ag/AgCl) to properly measure the potential at the working electrode, making the set-ups more complex compared to CCE. Alternatively, a standardized instrument could alleviate this inconvenience [199]. Apart from this, CPE is often accompanied by lower conversion rates than CCE. With controlled potential experiments, the current in the cell decreases over time as a result of the depletion of the substrate at the working electrode. Under galvanostatic conditions, however, the current is kept constant [177]. As the substrate concentration decreases, the potential across the cell must increase in order to maintain a constant electric current. The increasing voltage that is applied now also consumes the current in side reactions originating outside the target voltage (overoxidation-overreduction, oxidation-reduction of solvent molecules, etc.). The compromise of controlled potential experiments is, as the current decreases, the decrease of reaction rate and thus consumption of the starting material. This usually makes it difficult to operate a potentiostatic experiment to completion. Additionally, the reaction times to realize full conversion increase immensely. Nonetheless, potentiostatic electrolysis consumes the current more efficiently. In summary, the overall judgement on whether to use CCE or CPE is a settlement between experimental simplicity vs. current efficiency and fast/complete conversion vs. chemoselectivity. Moreover, with controlled potential, it is important to know the active potential at the working electrode, rather than the applied voltage. Ohmic drop (ΔE_ohm_) or ohmic polarization or IR drop is the potential drop between the reference and working electrode in an electrochemical cell (Figure 14) [200]. Because of this voltage drop, a different potential is experienced at the working electrode. An extra reason why electrosynthesis could be carried out under galvanostatic control is because the potential between the reference electrode and working electrode is only measured rather than controlled. The ohmic drop is proportional to the distance between the two electrodes (d), the magnitude of the current (I) and inversely proportional to the conductivity of the solution (ρ). For this reason, in electrosynthesis, the voltage drop is generally counteracted by the addition of supporting electrolytes to increase the conductivity (ρ). While solving the challenge of the ohmic drop, it often introduces other problems. Separation of the products from these supporting electrolytes following the electrolysis entails a substantial drawback. With the exception of scenarios after which the electrolyte can be recuperated and reused, a substantial amount of waste is being generated. Additionally, the recovery is often arduous as the ionic salts are inherently very soluble in conventional solvents. As discussed in Section 2.4, Francke et al. tackled this problem by designing a multipurpose redox mediator by integration of the supporting electrolyte into the iodine(I) precursor [91]. By means of such a method, recovery and reuse of both mediator and electrolyte becomes possible in a single step. Other approaches to reduce electrolyte usage generally often also rely on multifunctional reaction components. In the Kolbe electrolysis for example, the carboxylic acid substrate together with a base will form carboxylate salts that function as substrates as well as electrolytes. Following reaction completion, the base can simply be removed (e.g., filtration, extraction). Tajima, Fuchigami and co-workers showed that also solid-supported bases could serve as a similar solution by in situ generation of the electrolyte from methanol or HFIP as multifunctional solvents [201,202,203,204]. Ionic liquids can also be used as the solvent as well as the electrolyte in electrosynthesis [205,206].

As mentioned, ohmic drop is also subject to the distance between the working and reference electrode. The smaller the electrode gap, the lower the voltage drop in the electrolyte will be. The application of an electrochemical flow microreactor can dismiss the resistance associated with the electrolyte solution as it typically has a very small interelectrode distance [180,207]. For this reason, microflow electrolysis becomes possible in dilute electrolyte solutions or even obviates the need for supporting electrolytes, depending on the innate conductivity of the solvent system. Additionally, constant potential electrolysis becomes more feasible compared to batch, as good selectivity can still be accompanied by good conversion rates as well as high control of the degree of functionalization. A substrate’s degree of functionalization can be manipulated by managing the number of electrons supplied (F/mol).

Similar to how ohmic drop impedes electrosynthesis, the Beer–Lambert–Bouguer law limits photochemical transformations (Figure 14). As the attenuation effect of photon transport—or absorption (A)—is proportional to the penetration depth (d), photochemical reactions only occur in a limited film (millimeter magnitude) on the outside of the reactor [208,209,210,211]. This limited transmittance of irradiation due to absorption results in extended reaction times, especially in batch. This is a serious problem, and probably the biggest criticism on photoredox catalysis from a process chemistry point of view. Comparing photoredox catalyzed and electrochemical transformations in organic synthesis in this review, two observations are that generally the reaction times are longer and/or the scale in which they are performed are smaller for the photochemical approaches in comparison to the electrochemical. This is most likely a result of the limitation that the Beer–Lambert–Bouguer law sets. Standard upscaling of batch reactions will decrease the surface-to-volume ratio, which in turn will lengthen reaction times to impractical scale and increase the probability of side reactions. Nonetheless, scale-up of photoredox catalyzed reactions is possible, as displayed in Section 2.10 [165,166] and elsewhere [209,210]. These issues can be circumvented by the same technology electrosynthesis uses to cope with ohmic drop. Continuous-flow reactors have good surface-area-to-volume ratios and allow for more efficient irradiation of the reaction mixture opposed to a dimension-enlarging strategy for scale-up [208,209,210,211,212]. Scalability of electrochemical transformations is possible in batch (Section 2.10) as the addition of supporting electrolytes can counteract ohmic voltage drop. However, this is not an ideal solution because of the associated cost, substantial source of waste and obstacles with purification (vide supra). Flow microreactors can alleviate these issues for electrochemical methodologies as well [207].

### 3.3. Remoteness of Redox Events and Overall Redox Demand of Transformations

**Figure 15:**
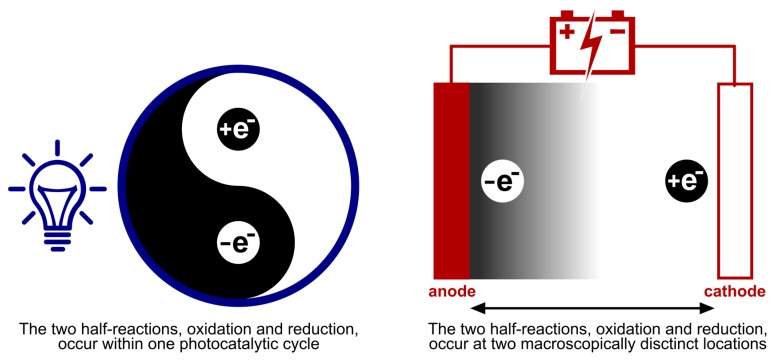
Visualization to emphasize the difference in remoteness of redox events between photoredox catalysis and electrochemistry.

Even though the gap between the anode and the cathode can be significantly reduced, the anodic oxidation and cathodic reduction are still separated on a macroscopic level. With photoredox catalysis, a homogeneous photocatalytic system allows for oxidation and reduction events to happen uniformly. This has significant consequences on the different types of redox transformations. In this section we will differentiate between net oxidative, net reductive and redox-neutral transformations. Net oxidative redox reactions are transformations for which an electron acceptor is required to act as the terminal oxidant. The opposite is true for net reductive redox reactions, demanding an electron donor as the terminal reductant. Redox-neutral transformations are characterized to have both oxidation and reduction steps involved in the same mechanism while retaining overall redox neutrality. From a mechanistic point of view, this can be more arduous than net oxidative or reductive transformations because both donation and acceptance of electrons is necessary at distinct positions in the reaction mechanism. For this reason, it is more facile for photoredox catalysis to execute and manage overall redox-neutral transformations than electrochemistry can. Due to the short-lived nature of the excited state photocatalyst and quenched, redox-active counterpart, both oxidants and reductants will be transiently present in the same reactive system. This temporal and disperse presence of both electron donors and acceptors allows for redox-neutral reactions to be possible. This is an advantage that photoredox catalysis has over methods utilizing stoichiometric chemical oxidants and reductants, as they are typically incompatible with one another. Achieving redox-neutral transformations via an electrochemical approach is very challenging as well because the oxidation and reduction events are macroscopically separated (mm-cm) due to the gap between anode and cathode, respectively. In Section 2.6, a redox-neutral transformation was displayed by both photoredox and electrocatalysis. Nonetheless, for the electrochemical method, a stoichiometric sacrificial HAT reagent is used to afford the final product instead of employing a catalytic HAT source in the photoredox process. This demonstrates that photoredox catalysis is more capable of taking advantage of the redox-neutral nature of such transformations. The two half-reactions occur within the same catalytic cycle for photoredox catalysis, while the oxidation and reduction half-reactions take place at remotely distinct locations in the electrochemical approach (Figure 15). As a result, most electrochemical transformations reported this far are net oxidative and net reductive in nature (with the majority anodic oxidations) [3,4,5]. In these methodologies, typically only one of the half-reactions affects substrates of interest. Yet, when the transformation is executed at the working electrode, the inverse half-reaction simultaneously occurs at the counter-electrode. In most of the recent approaches, this implicates the ineffectual redox conversion of solvent, electrolyte or other sacrificial species. To maximize energy efficiency, both electrodes could be engaged by executing two desirable processes simultaneously [3,213]. This is called paired electrolysis and allows valuable products to be formed at both electrodes.

Understanding their underlying physicochemical structure, it makes sense to, figuratively speaking, have these systems run on their own “redox-fuel”, to maximize energy efficiency and minimize waste production. This entails keeping the photoredox catalytic cycle a closed circuit, without adding external oxidants or reductants. The electrochemical half-reactions are ideally both benefitted from, to afford valuable products at both electrodes (paired electrolysis). This implies that photoredox catalysis is ideal for conducting redox-neutral transformations due to the homogeneous presence and transient nature of the redox-active excited photocatalyst and complementary oxidized/reduced state. Electrochemistry shows its full potential in net oxidative and net reductive transformations as electrons are inherently clean, renewable and inexpensive reactants. The adoption of electric current circumvents the use of conventional chemical oxidants and reductants and the waste source that goes along with them, increasing atom economy. Consequently, electrosynthesis can be used as a methodological strategy towards green chemistry [213,214]. This does not preclude photoredox catalysis from being a viable option towards green net oxidative or reductive transformations. Due to the external energy input via light irradiation, the terminal oxidant or reductant can be exceedingly mild in nature. A good example is the use of oxygen as the sole terminal oxidant where the photocatalyst acts as the active oxidant (Section 2.2, Section 2.3, Section 2.4, Section 2.5, Section 2.8, Section 2.10). They serve as mild alternatives to transformations that would otherwise need strong, waste-intensive oxidants. Moreover, similar to electrons, photons are readily available, clean and renewable reagents for organic synthesis. Due to the nature of the energy operated on, moving towards a future with renewable energy will cause both electrochemistry and photoredox catalysis to become intrinsically even greener.

### 3.4. Product Scope, Reactivity, Selectivity and Functional Group Tolerance

When both photoredox catalyzed and electrochemical methodologies exist for the same transformation, similarities in scope are often found, yet not trivially so. Opposite functional group tolerance and reactivity were also showcased in this review. As discussed above, electrochemistry and photoredox catalysis often compare very well to each other regarding their mode of activation. This can result in analogous reactivity, selectivity and functional group tolerance as extensively reviewed in Section 2 (Side by Side Comparison of Synthetic Methodologies). Conversely, opposite reactivity and functional group tolerance were highlighted as well. A commonality among these latter examples is the different—or absent—nature of a co-catalyst, when comparing both methodologies. For example, it is hardly surprising that visible-light mediated palladium co-catalysis leads to different reactivity than mediated electrosynthesis (Section 2.3 and Section 2.4). Similarly, the dehydrogenative lactamization performs better on diversely substituted amides, depending on the mode activation of the N-H bond (Section 2.2). The activation via the discussed PCET approach performs best on *N*-aryl benzamides, while the electrosynthesis of lactams was most productive for the *N*-acyloxy species. Additionally, the synthesis of complex quinolinone derivatives from *N*-phenyl cinnamamides was only possible via the photochemical approach due to the intrinsic added value it provides, being *E*/*Z* isomerization. Opposite functional group tolerance and reactivity was also observed for the decarboxylative sulfonylation (Section 2.5). Again, the involvement of an additive (KI) in the proposed mechanism could possibly explain this.

Many discussions that focus on achievable product scope have been discussed in the paragraphs above, as they are often innately linked to their modes of activation and restrictions. For the majority of the transformations discussed in this review, photoredox catalysis displayed either similar or superior selectivities in comparison with the electrochemical method. The only exception in this investigation is the alkyne oxygenation (Section 2.8). It is conceivable that an overarching explanation to this phenomenon exists, yet at this point, the coincidence hypothesis cannot be unambiguously discarded. A larger study would be necessary to back this claim, though in their current development, electrochemical methods appear to deliver faster and more robust substrate conversions compared to photoredox catalysis, which in turn demonstrates a more mild and selective reactivity profile.

## 4. Conclusions

In this review, we summarized and discussed 10 transformations that were performed by both photoredox catalysis and electrochemistry, and one photoelectrochemical method integrating these two. The two methodologies were subjected to a comparative investigation concerning the general reaction conditions, mechanism and product scope. The findings from these individual comparisons were compiled and used as basis for analysis to investigate where both research domains interlock and where they differ. Analogies were drawn between both methods to get a better understanding of both areas of research. While this survey does not indicate an overall preference for either of the two synthetic approaches, it does demonstrate the situations in which they manifest authority or restrictions over the other. In particular, a methodological design can be built around the approach that is theoretically favorable in terms of the overall redox demand of the transformation under investigation. Conceptually, photoredox catalysis is generally preferable for redox-neutral transformations. Conversely, electrochemistry excels when net reductive and oxidative transformations are contemplated. Nonetheless, it was clearly shown that both methods are able to bring about the same synthetic transformation, for which a very similar mode of activation is often the main reason. Due to this fundamental resemblance, this study also serves to provide a tool for the design of novel synthetic methodologies in both research areas, starting from previous knowledge of the other. However, we realize a case-by-case approach is still necessary. Moreover, appreciation of the underlying physicochemical and mechanistic structures will help to refine and innovate these two disciplines of organic synthesis. Additionally, an example was displayed where the combination of photoredox catalysis and electrochemistry leads to a constructive merger, where the shortcomings of one technique are complemented by the other. We believe this holistic effect has the potential to drive a powerful interdisciplinary addition to the synthetic toolbox.

Both methodologies are still facing, often similar, challenges. Going forward, we recognize that both research areas will have to solve similar problems in response to these challenges. The adoption of electrons and photons as readily available, clean and renewable reagents circumvents the use of conventional chemical oxidants and reductants and their concomitant waste. Consequently, photoredox catalysis and electrosynthesis can offer mild, alternative methodological strategies towards green chemistry.
